# Supplementary material for: The interactome of intact mitochondria by cross-linking mass spectrometry provides evidence for coexisting respiratory supercomplexes
Source: Mol Cell Proteomics. 2017 Dec 8;17(2):216–32. doi: 10.1074/mcp.RA117.000470 (PMC5795388; doi:10.1074/mcp.RA117.000470)
Supplement: Supplemental Data [file supp_RA117.000470_133922_0_supp_23978_fzffwf.zip › spectra_annotation/mito_spectra_annotation/440-1-1-1-5-1.pdf]

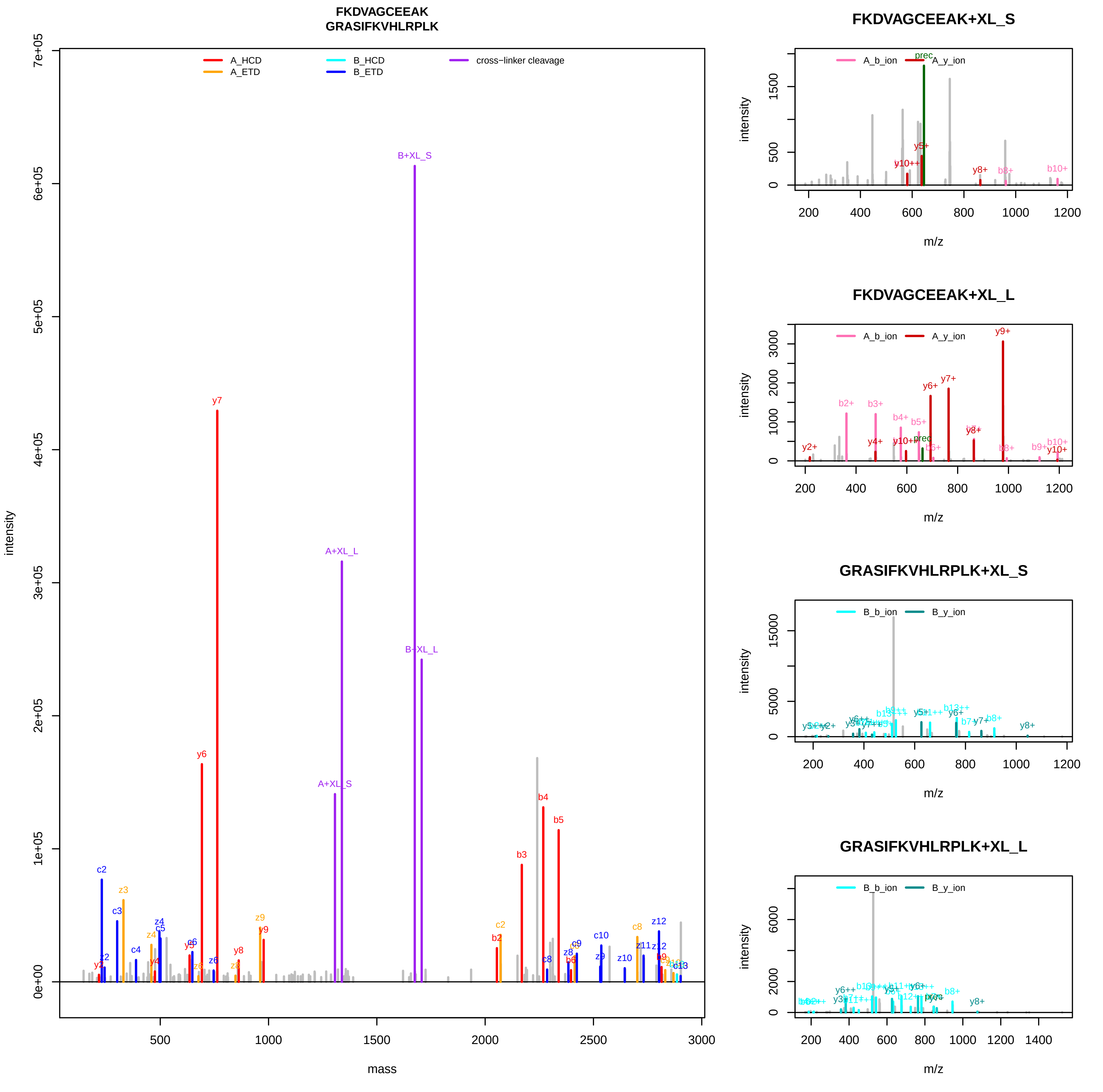
FKDVAGCEEAK
GRASIFKVHLRPLK
A_HCD
A_ETD
B_HCD
B_ETD
cross−linker cleavage
B+XL_S
A+XL_L
B+XL_L
A+XL_S
y7
y6
b3
b4
b5
b2
c2
c3
z3
z4
c4
c5
y4
y5
c6
z6
y8
z8
z9
y9
c8
c9
z8
c10
z10
c8
z11
z12
c13
intensity
mass
0e+00
1e+05
2e+05
3e+05
4e+05
5e+05
6e+05
7e+05
500
1000
1500
2000
2500
3000
FKDVAGCEEAK+XL_S
A_b_ion
A_y_ion
prec
y5+
y10++
y8+
b8+
b10+
intensity
m/z
FKDVAGCEEAK+XL_L
A_b_ion
A_y_ion
y9+
y7+
y6+
b2+
b3+
b4+
b5+
y8+
y2+
y4+
y10++
prec
b6+
b8+
b9+
b10+
y10+
intensity
m/z
GRASIFKVHLRPLK+XL_S
B_b_ion
B_y_ion
b13++
y6+
b7+
y7+
b8+
y8+
intensity
m/z
GRASIFKVHLRPLK+XL_L
B_b_ion
B_y_ion
y6++
y6+
b8+
y8+
intensity
m/z
